# The Effects of Acute Neutrophil Depletion on Resolution of Acute Influenza Infection, Establishment of Tissue Resident Memory (T_RM_), and Heterosubtypic Immunity

**DOI:** 10.1371/journal.pone.0164247

**Published:** 2016-10-14

**Authors:** Emma C. Reilly, Kris Lambert-Emo, David J. Topham

**Affiliations:** 1 David H. Smith Center for Vaccine Biology and Immunology, University of Rochester Medical Center, Rochester, New York, United States of America; 2 Department of Microbiology and Immunology, University of Rochester Medical Center, Rochester, New York, United States of America; University of Iowa, UNITED STATES

## Abstract

After disease resolution, a small subset of influenza specific CD8^+^ T cells can remain in the airways of the lung as a tissue resident memory population (T_RM_). These cells are critical for protection from subsequent infections with heterosubtypic influenza viruses. Although it is well established that expression of the collagen IV binding integrin alpha 1 is necessary for the retention and maintenance of T_RM_ cells, other requirements allowing them to localize to the airways and persist are less well understood. We recently demonstrated that inhibition of neutrophils or neutrophil derived chemokine CXCL12 during acute influenza virus infection reduces the effector T cell response and affects the ability of these cells to localize to the airways. We therefore sought to determine whether the defects that occur in the absence of neutrophils would persist throughout resolution of the disease and impact the development of the T_RM_ population. Interestingly, the early alterations in the CD8^+^ T cell response recover by two weeks post-infection, and mice form a protective population of T_RM_ cells. Overall, these observations show that acute neutrophil depletion results in a delay in the effector CD8^+^ T cell response, but does not adversely impact the development of T_RM_.

## Introduction

Tissue resident memory CD8^+^ T cells (T_RM_) comprise a distinct immune population that remains localized to the area of infection after resolution of a disease in peripheral tissues[1,2]. T_RM_ cells are uniquely poised to respond to subsequent pathogen challenges and upon re-exposure to the infectious agent, this T cell subset truly represents the first line of cellular immune defense; mounting a response as early, if not prior to, that of the innate immune system[3]. Mice lacking resident memory T cells quickly become susceptible to lethal secondary challenge, highlighting the importance of this rapid, local response, and rendering T_RM_ cells indispensible[4,5].

T_RM_ cells are present in a number of non-lymphoid tissues, including the skin, the salivary glands, the gut, brain, uterus, and notably, in the context of influenza virus, the lungs[6–12]. Despite similarities in function, development and maintenance requirements for the different T_RM_ populations vary between tissues. For example, in the gut, the expression of integrin α4β7 (LPAM-1) and CCR9 are required on the T cells for homing to the intestines[13–15]. Alternatively, CLA and CCR4 promote entry into the skin while IL-15 and IL-7 are important for sustaining the population in the tissue[16–19]. Interestingly, despite the fact that many of the seminal studies of T_RM_ cells originated within the influenza field, there is an incomplete understanding of the critical mediators of development and persistence of the population.

Expression of CXCR3, the receptor for chemokines CXCL9 and CXCL10, which are made early during influenza virus infection, has been implicated in the generation of airway memory, however, this was in the context of vaccination and not natural infection[20,21]. In fact, other studies suggest that the role of CXCR3 is in the development of short-lived effector CD8^+^ T cells and that the memory population is better sustained in the absence of this receptor [22,23]. Other potential requirements for the maintenance of pulmonary T_RM_ cells include the expression of tissue binding integrins and activation markers, which promote retention. Lung T_RM_ cells express integrins α1β1 (VLA-1), αE (CD103) paired with integrin β7, and CD69, with the latter two considered the prototypical T_RM_ markers in other tissues[5,24,25]. Integrin αE (CD103)/β7 is the receptor for epithelial cell tight junction protein E-cadherin, suggesting expression of this integrin could allow for direct interaction of the cells with the tissue[26]. CD69, is an inhibitor of sphingosine 1-phosphate receptor 1 (S1P1), and has been shown to limit egress of cells from both lymphoid and non-lymphoid organs[27–29]. High expression of CD69 on pulmonary T_RM_ cells would therefore improve retention within the lungs. Elimination of either of these molecules results in a reduction in CD8^+^ T cell accumulation and retention after infection, implying that both play important roles, but neither is sufficient to completely ablate the development of a memory pool[25]. Integrin α1 (CD49a) is a collagen binding integrin, with preference for collagen IV; one of the main constituents of the lamina densa underlying the epithelium[30,31]. Although less well defined in other organ systems, expression of integrin α1 is essential for maintenance of CD8^+^ T_RM_ cells in the lung[5]. Absence of integrin α1 results in a significant decrease in the T_RM_ population and increases susceptibility to secondary influenza virus infection. This requirement for expression of a collagen binding integrin further supports the notion that cell-tissue interactions facilitate maintenance of T_RM_ cells.

The lifespan of T_RM_ cells is also location dependent. Both skin and intestine resident memory cells have been shown to self renew, which could be a result of constant antigen exposure from the commensal microbiota or exposure to the local cytokine milieu[4,32,33]. In contrast, CD8^+^ T_RM_ cells in the lung have a limited half-life of only 6 months[4,34]. The short-lived nature of this population results in susceptibility to reinfection with similar viruses. Therefore, it is essential to gain more insight into which cues are required as well as those that are redundant or not essential for the establishment and maintenance of resident memory cells in the lungs.

Recent studies have revealed that neutrophils can support effector CD8^+^ T cell migration and function during influenza virus infection through the release of CXCL-12 rich membrane vesicles[35,36]. In the absence of these granulocytes, virus specific CD8^+^ T cells accumulate to a lesser extent during the acute phase of infection and are positioned distal to the airway compared with non-depleted controls. This would suggest that CD8^+^ T cells do not adequately reach the infected epithelium to eliminate the virus in the absence of neutrophils. This defect in trafficking could therefore be responsible for the observed delay in clearance of influenza virus and may limit the accumulation of airway-associated memory.

The dependence on neutrophil derived CXCL-12 demonstrates a clear requirement for chemokine-mediated migration and/or activation during the acute response. However, it remains unclear whether this early impediment in the effector CD8^+^ T cell response is durable, and if these early changes can significantly alter the T_RM_ population localized to the airways. Therefore, we set out to evaluate the CD8^+^ T cell responses through the acute, resolution, and memory phases of influenza virus infection to ultimately determine whether the absence of neutrophils during acute primary infection, and the resulting impairment in CD8^+^ T cell trafficking, impact the development of long-lasting local memory.

## Materials and Methods

### Mice

All mice were maintained and managed in university approved, pathogen free facilities using microisolator technology. C57BL/6 mice were purchased from Jackson Laboratories (Bar Harbor, ME). All mice were 8–10 weeks at the start of the infection. A colony of OT-1 transgenic mice that express a TCR specific for the OVA SIINFEKL (OVA_257-264_) peptide presented in the context of H-2K^b^ were crossed with a transgenic mouse expressing GFP under a β-actin promoter. This study was carried out in strict accordance with the recommendations in the Guide for the Care and Use of Laboratory Animals as defined by the National Institutes of Health. Animal protocols were reviewed and approved by the Institutional Animal Care and Use Committee (IACUC) of the University of Rochester. All animals were housed in a centralized and AAALAC-accredited research animal facility that is fully staffed with trained husbandry, technical, and veterinary personnel.

### Treatment and cell transfer

For neutrophil depletion, mice were sedated on day -2 with avertin (2,2,2-tribomoethanol) and given an intranasal (IN) inoculation of 200μg anti-Ly6G antibody (clone 1A8) or IgG control antibody (clone 2A3) in 30μL volume. Mice were also given 200μg antibody IP in 200μL volume. IP injections were subsequently given on days -1, 1, 3, 5, and 7 post-infection. For imaging experiments, 1x GFP OT-1 cells were transferred intravenously by tail vein on day -1 to C57BL/6 hosts.

### Viruses and Infections

The influenza H3N2 A/Hong Kong/X31 (X31) virus, A/X31-OVA-1 influenza virus that expresses the ovalbumin (OVA257-264 SIINFEKL) peptide in the hemagglutinin viral protein, and H1N1 A/Puerto Rico/8 (PR8) were grown and titered in embryonated chicken eggs and harvested as allantoic fluid preparations. Mice were sedated with avertin and infected IN with 10^5^ EID50 of X31, 3x10^3^ EID_50_ X31-OVA- 1, or 10^3^ EID_50_ PR8 in 30μL of PBS. After infections in all experiments including survival studies, mice were monitored daily for weight loss, ability to ambulate, ability to intake food and water, and signs of discomfort including ruffled fur, hunched posture, and guarding behavior. As a humane endpoint, mice that lost ≥30% of their body weight, or exhibited other signs of undue discomfort, were euthanized using an overdose of the anesthetic avertin given as an intraperitoneal injection, followed by secondary cervical dislocation. Despite daily monitoring, some mice were found post-mortem, and immediately removed from the cage to minimize distress of the remaining mice.

### Cellular Preparations

Bronchoalveolar lavage (BAL) samples were obtained by cannulating the trachea and flushing lungs with 1X PBS. Cells were spun down and lysed with 500μL of ACK lysis buffer (Ammonium-Chloride-Potassium) for 5 minutes at room temperature. Cells were washed in PBS containing 1% FBS (PBS serum) and resuspended for counting in 1mL of PBS serum. Lungs were perfused with 1x PBS, removed, and separated into right and left lobes. Lung tissue was dissociated in C tubes by the GentleMACS (Miltenyi Biotek) using the Lung01 program. Samples were incubated in 5mL [2μg/mL] Collagenase II in RPMI +8% FBS at 37°C for 30 minutes, with gentle agitation every 10 minutes. After digestion, samples were further dissociated using the Heart01 program. Cell suspensions were filtered through 100μm filters prior to 75:40 Percoll (GE Healthcare) discontinuous gradient separation. The top layer, containing fat and other debris, was removed by aspiration. The cell layer was harvested and washed, prior to counting and staining. Counting was achieved through Trypan blue exclusion on a hemocytometer.

### Flow cytometry

Single cell suspensions were stained in PBS containing 1% FBS, purified CD16/32 (clone 2.4G2), and some combination of the following antibodies: TCRβ, CD8α, CD69, CD49a, CD103, CD44, CD62L, Gr-1, and CD11b. For intracellular staining, the following antibodies were used: Lamp1, TNFα, IFNγ, Granzyme A, and Granzyme B. Lamp1 was added for 4 hours during *in vitro* culture. The remaining intracellular antibodies were added after fixing and permeabilization with the BD Biosciences intracellular staining kit. All antibodies were obtained from either eBioscience or BD Biosciences. NP tetramer was obtained from the NIH tetramer core facility (Atlanta, GA). Cells were analyzed by LSRII (BD Biosciences) in the University of Rochester Flow Cytometry core facility and analyzed using FlowJo software (Tree Star).

### Cytospin

A glass slide and a single Cytofunnel were placed into a Cytoclip for each sample (Thermo Scientific Shandon). 3x10^4^ cells were added in a volume of 100μL PBS to each funnel. Clips were placed in the Cytospin 2 (Thermo Scientific Shandon) and run at 1,000rpm for 5 minutes. Slides were removed and allowed to dry at room temperature. Diff Quick stain was used to stain slides (Siemens) prior to counting on a light microscope.

### Multiphoton Imaging

Imaging was performed as described in Lambert-Emo, *et al*.[37]. Briefly, images were captured using an Olympus FV1000AOM-Multiphoton imaging system in combination with a Spectra-Physics MaiTai-HP Deep See fs Ti:Sa laser system in the University of Rochester Light Microscopy core. Prior to surgery, animal was anesthetized with pentobarbital [65 mg/kg]. Surgical procedure exposed and separated the trachea from the surrounding tissues, and a cannula was inserted to provide oxygen and 0.5% isoflurane throughout imaging session. The animal was attached to a pre-heated stage, and temperature was maintained at 37°C using a heating blanket with feedback from a rectal probe. Additionally, physiological stats were measured using a MouseOX Plus sensor (Starr Life Sciences). Once the animal was stable, reflexes were used to verify sufficient sedation, and Pancuronium bromide [0.4mg/kg] was administered prior to imaging, to prevent movement of the imaging area. All imaging was done with a heated 25x objective at 256 pixel resolution. All imaging data were analyzed and visualized using Imaris (Bitplane) software.

### *In vitro* stimulation

BAL cells were plated 1x10^5^ cells per well in RPMI serum (8%FBS) in a 96-well plate. NP_(366–374)_ was added at [1μM]. As a positive control, cells were crosslinked with plate-bound CD3/CD28 antibodies. Incubation with media alone was used to determine baseline levels of cytokine production. Cells were incubated at 37°C. After 2 hours of incubation, anti-Lamp1 antibody and GolgiPlug [1μL/mL] were added to the wells, and cells were incubated for 4 more hours prior to harvest and intracellular staining.

### Viral titers by Immunofluorescence Assay

MDCK cells were plated in a 96-well tissue culture dish to reach confluence within 24 hours. Samples were diluted in a separate 96-well plate. 100μL Lavage fluid was added to the top row of wells in triplicate. 90μL of Virus Dilution Media (1X PBS, 0.3% BSA, 1% pen/strep) was added to remaining wells. 10 fold dilutions were achieved by transferring 10μL to wells in subsequent rows, changing tips prior to further dilution. PR8 virus stock was used as a positive control and media alone was a negative control. Media was removed from MDCK cells and cells were washed 2X with PBS. 50μL of virus samples from the dilution plate were added to the cells. Plate was incubated at room temperature for 1 hour with gentle rocking every 15 minutes. Viral samples were aspirated from the cells. 100μL of warmed PI MEM (DMEM, 0.3% BSA, 1% Pen/Strep/Glut, TPCK Trypsin) added to each well. Plate incubated 8–10 hours at 37°C. PI MEM was aspirated from the wells and 100μL fixative added (1:1 Methanol:Acetone). Plate incubated for 20 minutes at -20°C. Wells were washed 2X with PBS. 100μL PBST (1% BSA, 0.1% Tween20) added per well. Plate was incubated for 45 minutes at room temperature. PBST was removed and primary anti-influenza NP antibody (EMD Millipore) added 1:1000 in PBS containing 1% BSA. Plate incubated 90 minutes at room temperature. Plate washed 3X with PBS. Secondary antibody (Goat anti-mouse AF488) (Life Technologies) was added 1:500 in PBS 1% BSA. The plate was incubated for 45 minutes at room temperature. Plate was washed 3X in PBS. 100μL PBS added per well. Plates were quantified by manual counting of fluorescent plaques.

### Statistics

All statistics were performed using Prism Analysis Software (GraphPad). When data was analyzed within a time course, a two-way ANOVA, followed by post-hoc T-tests were used. For two groups at a single time point, which were equally distributed, an unpaired Student’s T-test was performed. For imaging data which cannot be assumed to be normally distributed, a Mann-Whitney Test was performed. Significance was considered a p-value <0.05.

## Results

### Neutrophil depletion during acute influenza infection of the lung

We have previously shown that depletion of neutrophils during acute influenza infection limited CD8^+^ T cell infiltration on day 7 of infection and delayed viral clearance[35]. The effects on the CD8^+^ T cell response at other time points were not measured. We therefore decided to look at additional time points. To be certain we were depleting the neutrophils, a regimen using an antibody specific for Ly6G (clone 1A8) both intranasally and intraperitoneally was implemented (S1 Fig). To ensure that this method was effective and eliminated >90% of neutrophils, lung samples were evaluated at day 6 post-infection both by flow cytometry and cytospin. High levels of Ly6G and CD11b distinguish neutrophils from other granulocyte and myeloid populations. However, since an antibody against Ly6G was used for the depletion, a different clone, which binds both Ly6C and Ly6G (Gr-1), was used to separate the population from monocytes. By flow cytometry, the population of Gr-1 and CD11b high expressing cells was diminished (S1 Fig). The absence of neutrophils was further confirmed by loss of a population of large cells with high levels of internal complexity as measured by forward and side-scatter parameters. When quantified, the remaining neutrophils accounted for less than 10% of the population in control mice, which is consistent with previous reports (S1 Fig). To conclusively verify that the observed changes were not due to down regulation of surface expression of these markers or alterations in size and surface properties, lung tissue samples were analyzed by cytospin (S1 Fig). Similar to flow cytometry data, cytospins showed a substantial (>90%) reduction in the neutrophil population after treatment with anti-Ly6G. Thus we were confident that neutrophils were being adequately depleted.

### Effects of neutrophil depletion on the kinetics of the CD8^+^ T cell response in the lung

Elimination of neutrophil derived CXCL-12 resulted in a decrease in the magnitude of the virus- specific CD8^+^ T cell response on day 7 of the effector response[35]. However, it was not previously determined if this CXCL-12 dependency would persist throughout all stages of influenza infection, including the formation and maintenance of local memory. To address whether neutrophil depletion resulted in a persistent decrease in the acute CD8^+^ T cell response, it was examined prior to infection (day 0), during the peak of infection (days 6 and 8) and after clearance of the virus (day 14). Lung parenchymal CD8^+^ T cells were recovered through mechanical disruption of the tissue followed by collagenase digestion and airway CD8^+^ T cells were harvested through bronchoalveolar lavage (BAL). Flow cytometric analysis was used to identify a population of classical CD8^+^ T cells (TCRβ^+^CD8α^+^).

**Fig 1:**
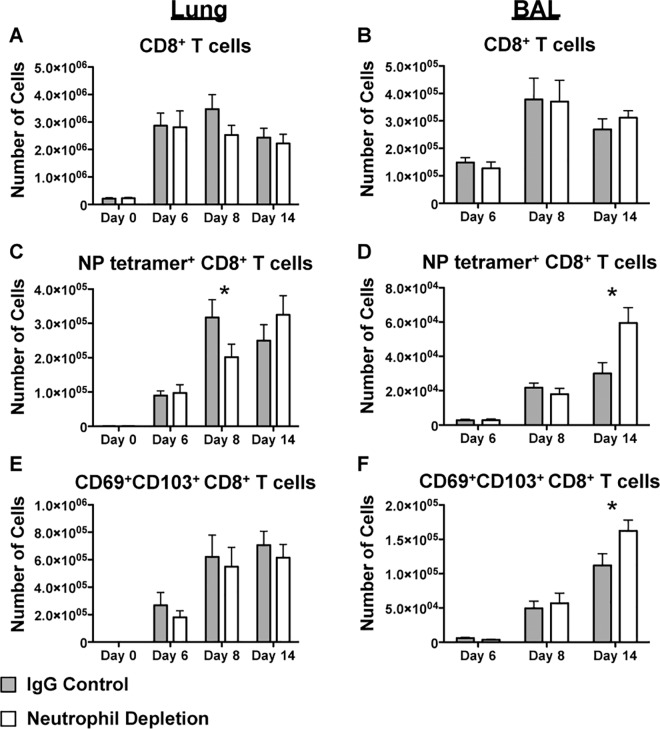
The virus-specific CD8^+^ T cell response is delayed in the absence of neutrophils. CD8^+^ T cells from the lung parenchyma (Lung) and airways (BAL) on days 0, 6, 8, and 14 post-infection were evaluated by flow cytometry by staining for TCRβ and CD8α (A,B). NP tetramer^+^ (C,D) and CD69^+^CD103^+^ CD8^+^ T cells (E,F) were similarly quantified. Data is the combination of 3–4 experiments represented as mean ± SEM. * is p-value < 0.05 in two-way ANOVA followed by *post-hoc* t-test.

Consistent with previous work by Tate, *et al*., depletion of neutrophils by 1A8 did not impact the total number of CD8^+^ T cells (Fig 1A and 1B)[36]. To evaluate virus-specific cells, we focused on NP specific CD8^+^ T cells since we were predominantly interested in the generation and maintenance of memory cells, and NP specific cells account for 80–90% of the response in a secondary challenge[38]. No consistent significant differences were observed in the number of NP tetramer^+^ T cells in the lung (Fig 1C) or BAL (Fig 1D) at day 6 post-infection in neutrophil depleted mice compared with controls, which can likely be attributed to variability in the early adaptive response. At day 8 however, comparable to other reports at similar time points [35,36], significantly lower numbers of NP tetramer^+^ virus specific CD8^+^ T cells were found in the lung tissue, but not in the BAL (Fig 1C). While this may be counterintuitive given the changes we previously observed in the localization of CD8^+^ T cells distal to the airway in the trachea at a similar time point, it is consistent with data observed in Tate, *et al*. [36] suggesting the mechanism of recruitment to the airway is not fully understood.

Therefore, we next sought to determine whether CD8^+^ T cells could overcome these early, modest deficits in the response that we observed in the lung. Fourteen (14) days after infection, virus specific CD8^+^ T cells were present in comparable numbers in both groups in the lung tissue (Fig 1C). Additionally, an unexpected result was that the NP-specific population of T cells from BAL was enhanced in the neutrophil depleted mice, suggesting that neutrophil signals may support effective and efficient early killer T cell responses, but that later on, CD8^+^ T cells are capable of recovering from this initial delay (Fig 1D).

Given that NP tetramer^+^ CD8^+^ T cells at day 14 could still be residual effector cells rather than resident memory precursor cells, we also evaluated the expression of two of the prototypical T_RM_ markers, CD103 and CD69. Although there is no way of definitively separating T_RM_ precursors at this early time point from residual effectors, we believed that expression of these markers would promote the retention of these cells within the airways (Fig 1E and 1F). Predictably, we observed a similar increase in the CD103 CD69 double expressing CD8^+^ T cell population in the BAL, without a concurrent increase in the lung parenchyma. The data overall suggest that neutrophils are more important for early CD8^+^ T cell infiltration into the lungs, and at later stages of the response, T cell infiltration recovers.

### The effect of neutrophil depletion on CD8^+^ T cell motility in the trachea

**Fig 2:**
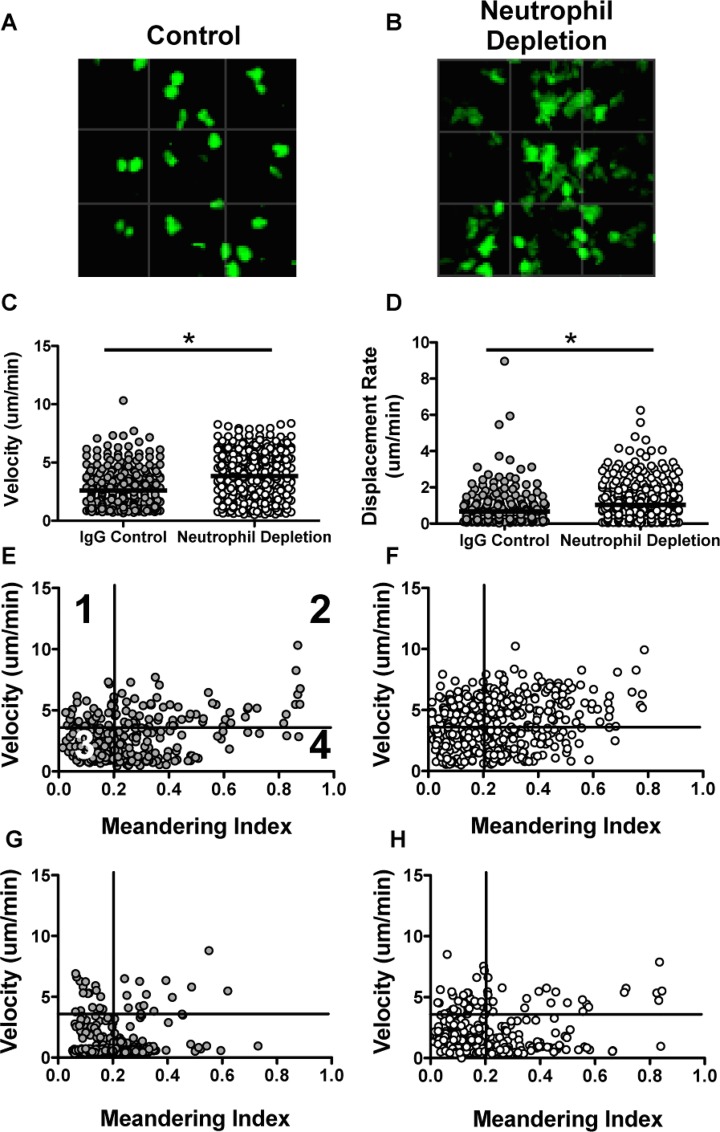
CD8^+^ T cells display altered migration at day 9 post-infection. GFP OT-1 cells were transferred into C57BL/6 recipients and mice were subsequently infected with HKx31 OVA influenza virus. Cells were imaged by multiphoton microscopy at days 9 and 10 post-infection. Representative images of GFP OT-1 cells at day 9 in control (A) and neutrophil depleted (B) mice. Graphs represent the velocity (C) and displacement rate (D) at day 9 post-infection, with each dot representing a single cell. Migration is also shown as velocity vs. meandering index at day 9 in control (E) and neutrophil depleted (F) mice and day 10 post-infection in control (G) and neutrophil depleted (H) mice[40]. Briefly, numbered quadrants as shown in E represent the following migratory patterns: 1) Active migration with return to origin 2) Directional, sustained motility 3) Low motility 4) Non-sustained migration. Migration parameters were analyzed by Imaris Imaging Software (Bitplane). * p-value <0.05 based on the Mann-Whitney test.

While our early analysis of the cellular phenotype throughout infection suggested that elimination of neutrophils resulted in a delay in the CD8^+^ T cell response, rather than sustained impairment, we were interested to examine whether the behavior of the cells *in vivo* would show a similar delay and recovery. In our lab, we previously established a method of imaging virus-specific CD8^+^ T cells within the trachea[37]. We recognize that some differences in the kinetics of clearing the infection may result in differences from those observed in the lungs, however, it is known that the virus is cleared from the organ by day 9 in wild type mice. It is also well established that in the absence of neutrophils, viral clearance is delayed compared with control mice[39]. Therefore, we used the intravital imaging setup to evaluate cellular migration patterns of CD8^+^ T cells at days 9 and 10 post-infection, to determine if we could see a similar recovery to what we observed *ex vivo*. In control mice at day 9, the CD8^+^ T cells appear small and round, and show a slow rate of displacement (Fig 2A and 2D). We found that virus-specific CD8^+^ T cells in mice depleted of neutrophils (Fig 2B) at this same time point display altered morphology (Fig 2B), as well as migration (Controls: Fig 2C–2E, S1 Video, Neutrophil Depleted: Fig 2C, 2D and 2F, S2 Video). CD8^+^ T cells in mice depleted of neutrophils appear larger in size and display increased velocity and displacement rates compared with control mice (Fig 2A–2D). Looking at plots of velocity versus meandering index (a measure of confinement), we were able to further infer migration habits of the T cells[40]. As shown in Fig 2E and Table 1, each numbered quadrant of the 2D plot represents a different type of movement. On day 9 the T cells in the neutrophil depleted mice (Fig 2F) demonstrated high velocities and low confinement compared with the control mice (Fig 2E), consistent with the notion that the mice display delayed elimination of virus and antigen. Interestingly, when we evaluated cell migration at day 10 post-infection, we observed similar migration parameters to control mice at day 9 post-infection, indicating that the CD8^+^ T cell responses are delayed, but most likely not deficient by this time point (Control: Fig 2G, Neutrophil Depleted: Fig 2H).

**Table 1:** Motility parameters by quadrant.

Quadrant 1:Velocity is high Meandering Index is low “Active migration, returns to origin”	Quadrant 2: Velocity is high Meandering Index is high “Directional, sustained motility”
Quadrant 3: Velocity is low Meandering Index is low “Low motility”	Quadrant 4: Velocity is low Meandering Index is high “Non-sustained motility”

### Development of T_RM_ in the lung after neutrophil depletion

**Fig 3:**
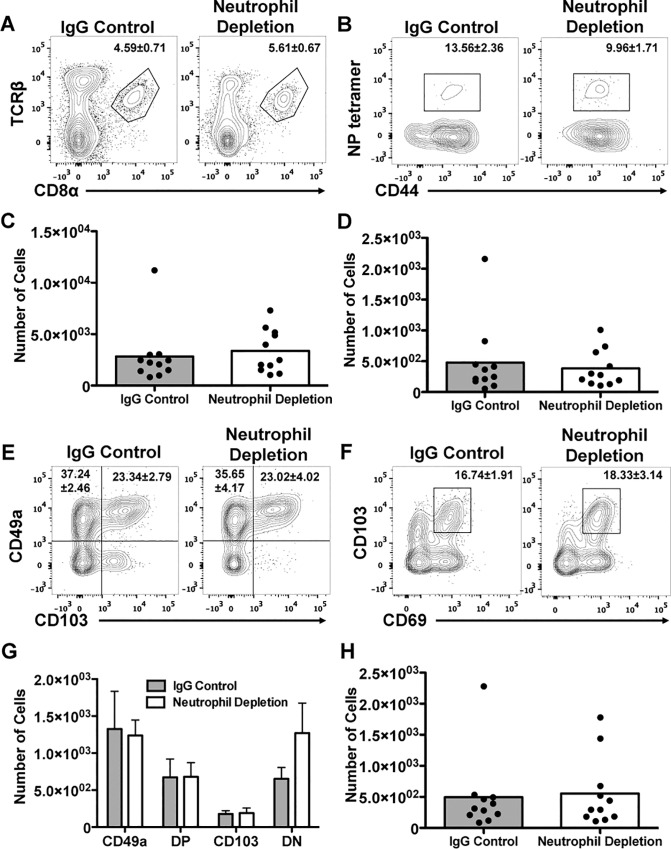
Depletion of neutrophils during acute infection does not limit the long-term accumulation of influenza specific, CD103^+^, CD69^+^, or CD49a^+^ CD8^+^ cells in the airways. At approximately 3 months post-infection, bronchoalveolar lavage (BAL) CD8^+^T cells (A,C) were analyzed for NP-specificity (B,D) and prototypical T_RM_ markers (E-H) by flow cytometry. Data shown is 3 separate experiments combined.

We had speculated in our earlier work that T_RM_ could be affected in neutrophil depleted mice[35]. However, based on the current observations (Fig 1), with the accumulation of virus-specific T cells at the end of the primary response, we anticipated that the development of the airway resident memory population would be uninterrupted, and possibly enhanced, in the absence of neutrophils. To determine this, mice depleted of neutrophils during a primary infection with HK-x31 influenza virus were rested for 3 months and then examined for the presence of T_RM_ cells in the airways. CD8^+^ T cell percentage and numbers in the BAL were determined by flow cytometry (Fig 3A and 3C). The whole CD8^+^ T cell population was a similar size regardless of neutrophil status. Similarly, comparable frequencies of virus specific cells and phenotypic T_RM_ cells as identified by expression of integrins α1, αE, and CD69 (Fig 3B and 3D–3H) were present. While T_RM_ cells in the airways have been shown to be the protective population, we also examined the CD8^+^ T cell population in the lung parenchyma, and found no differences between the groups (S2 Fig) [5,41]. Thus neutrophil depletion during the acute primary infection does not impair or enhance the formation of T_RM_.

### Function of T_RM_ cells *in vitro*

**Fig 4:**
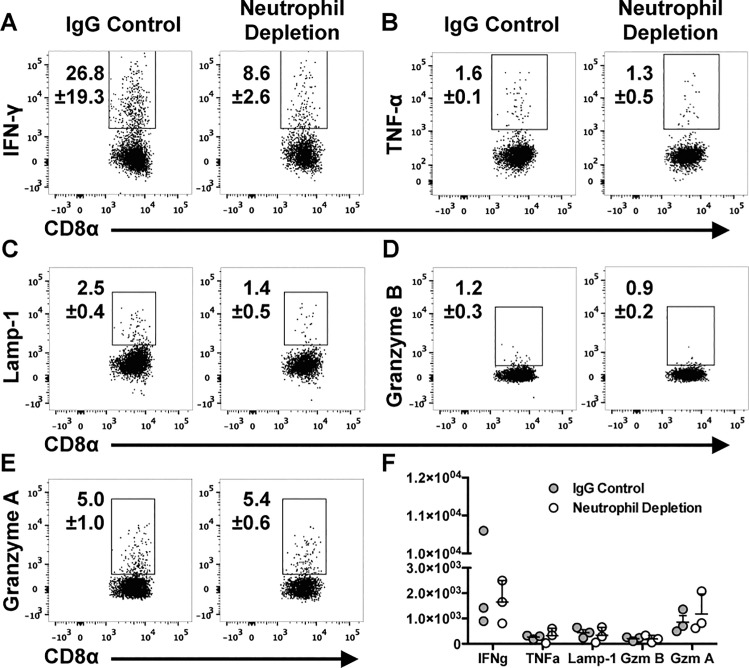
The ability of T_RM_ cells to produce cytokines and granzymes *in vitro* is not altered by acute neutrophil depletion. BAL cells were incubated *in vitro* with NP peptide for 6 hours and analyzed for IFN-γ (A), TNF-α (B), Lamp-1 (C), Granzyme B (D), and Granzyme A (E) by intracellular staining and flow cytometric analysis. The number of positive cells was quantified (F). Flow plots are a concatenation of 3 samples (A-E). In F, each point represents data from one mouse.

After identifying comparable populations of cells that are phenotypically defined as T_RM_ cells (expression of CD103, CD69, CD49a) in both airway and lung tissue, we asked whether these cells would display similar functional capabilities upon rechallenge. After *in vitro* restimulation with NP(366–374) peptide, BAL CD8^+^ cells from mice previously devoid of neutrophils produced IFNγ, TNFα, and displayed a marker of previous degranulation (Lamp1) (Fig 4A–4C). While neither group showed CD8^+^ T cells producing significant amounts of granzyme B (Fig 4D), both groups expressed granzyme A, and at similar levels (Fig 4E). Although the lung parenchyma contains a more diverse population of CD8^+^ T cells at this time point, by CD62L and CD44 expression (S3 Fig), CD8^+^ T cells in the lungs of both groups of mice displayed similar responses to stimulation with NP tetramer (S4 Fig). All together, no changes were observed between the groups of mice, suggesting that NP specific cells that form in the absence of neutrophils and remain in the airway or lung tissue have a similar functional capacity to those in control mice (Fig 4F and S4 Fig).

### Effect of neutrophil depletion on secondary immune protection conferred by T_RM_

**Fig 5:**
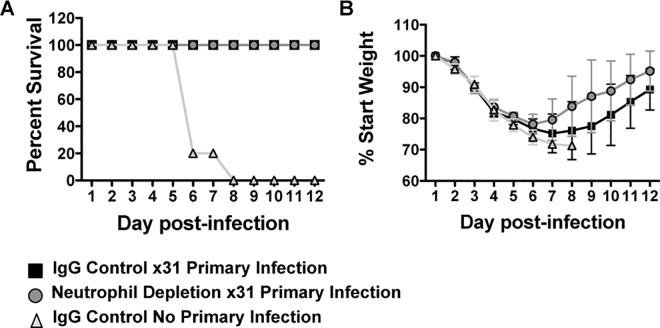
Mice depleted of neutrophils during primary influenza virus infection are not susceptible to lethal heterosubtypic challenge. Mice previously infected with HKx31 influenza virus with and without neutrophil depletion were rechallenged with a lethal dose of hetersubtypic virus, PR8 [1x103 PFU/mouse]. Mice were monitored for survival (A) and weight loss (B). Data is representative of 2 independent experiments with 5 mice per group. In B, data is shown as mean ± SD.

It is well established that T_RM_ cells are critical for surviving challenge with a lethal dose of heterosubtypic influenza virus[4,5]. In their absence, mice rapidly succumb to the infection even in the presence of other memory cell subsets (T_EM_ and T_CM_)[4,41]. Although we identified a population of cells that are phenotypically defined as T_RM_ cells and found the cells to be functionally equivalent *in vitro*, it did not elucidate whether they were protective *in vivo*. To address this critical question, upon recovery of the primary infection, mice were rested for three months to allow for generation of T_RM_, and challenged with a lethal dose of heterosubtypic virus, PR8 influenza. Mice were then monitored for weight loss as a measure of morbidity and survival. As a control, mice with no history of influenza virus infection were challenged with PR8. As anticipated, all mice with no previous exposure to influenza virus infection quickly succumbed to the PR8 challenge (Fig 5A). However, all of the remaining mice, regardless of neutrophil status during the primary infection, were protected (Fig 5A). Furthermore, the mice that previously lacked neutrophils had similar if not reduced morbidity during rechallenge (Fig 5B). These results show that the T_RM_ that develop in mice depleted of neutrophils during the priming phase can provide immune protection.

### Secondary Response of CD8^+^ T cells

**Fig 6:**
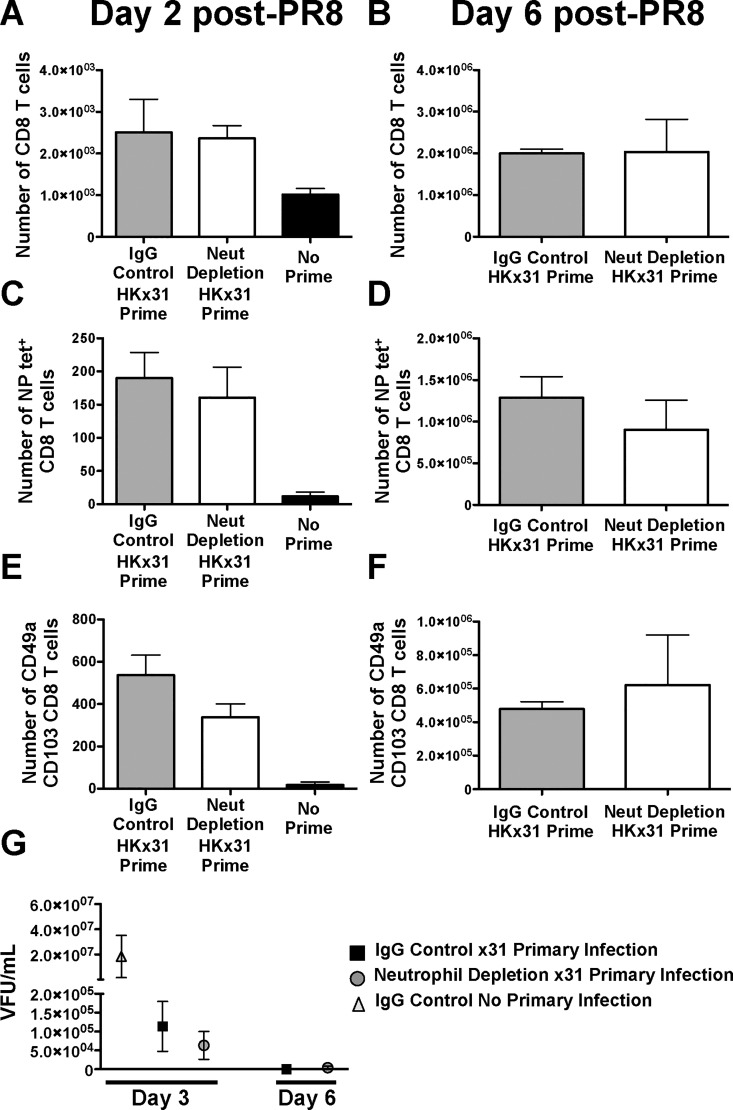
Mice depleted of neutrophils during primary influenza virus infection mount an effective secondary response to heterosubtypic challenge. Mice previously infected with HKx31 influenza virus with and without neutrophil depletion were rechallenged with heterosubtypic virus, PR8, and CD8^+^ T cell responses were measured by flow cytometry. Total CD8^+^ T cells (A,B), NP-specific CD8^+^ T cells (C,D), CD49a^+^ CD103^+^ CD8^+^ T cells (E,F) were measured at days 2 (A,C,E) and 6 (B,D,F) post-infection. Viral titers at days 3 and 6 were quantified by immunofluorescence assay (G). Data is from 3–6 mice and displayed as mean ± SEM.

Although depletion of neutrophils results in an impairment in local, acute CD8^+^ T cell responses, development of systemic memory is unaffected[36]. To verify this, mice were rechallenged with PR8 influenza virus at 3 months post X31 infection and examined for virus specific CD8^+^ T cell recruitment to the airways. At day 2 post infection, a time point prior to recruitment of central memory T cells, there are comparable numbers of total CD8^+^ T cells as well as NP specific cells (Fig 6A and 6C and S5 Fig). Similar to the observation prior to rechallenge, we also detect comparable frequencies of CD49a CD103 double positive cells (Fig 6E and S5 Fig) and CD103 CD69 coexpressing cells (S5 Fig). Recruitment of systemic memory cells does not appear to be interrupted as both groups of mice display similar numbers of virus-specific CD8^+^ T cells at day 6 post-infection (Fig 6B and 6D). Cells expressing CD49a and CD103/CD69 still remain comparable between the groups (Fig 6F and S5 Fig). Viral titers at early time points after rechallenge (day 3 and day 6) (Fig 6G) further confirm that acute neutrophil depletion during primary influenza virus infection does not impair the protective capacity of T_RM_ and other memory cell populations.

## Discussion

Effective antiviral responses require the coordination of both the innate and adaptive arms of the immune system. In the context of influenza virus, ultimate clearance of the virus is through cytotoxic CD8^+^ T cells[42,43]. However, as we previously described, elimination of neutrophils, and specifically neutrophil derived CXCL12, perturbs this response, and results in lower virus-specific CD8^+^ T cell numbers, localization distal to the airways compared to controls, and delayed clearance of virus[35]. In this study, we sought to determine if the early defects in CD8^+^ T cell responses persisted through disease resolution and had long-lasting consequences including impairment in the development of local memory.

The observation that virus-specific CD8^+^ T cells are compromised early during influenzavirus infection in the absence of neutrophils was consistent with previous reports[35,36]. However, we discovered that this defect recovered within two weeks after infection. In fact, at this time point, NP tetramer positive cell numbers were enhanced in the airways. Influenza virus infection in the absence of neutrophils results in increased inflammation, edema, and damage to the lungs, which could result in additional access to cells in proximity to the airways by bronchoalveolar lavage[39]. However, we identified comparable numbers of virus specific cells in the lung tissue suggesting our data reflect an overall resurgence in the population. Another possibility is that a secondary wave of neutrophils promotes later CD8^+^ T cell responses. Neutrophils are notoriously difficult to eliminate in their entirety, as they are continuously produced and replenished at high rates. In mice, under homeostatic conditions, <2.5 x 10^6^ neutrophils are circulating at a given time, though this number can increase 10-fold to combat infection or injury[44]. Another caveat is that although circulating neutrophils have a short lifespan of only 6–8 hours, longer-lived resident populations exist within the lungs[45]. However, we find this explanation to be unlikely since even though the depletion regimen was only carried out through day 7, the levels of neutrophils were significantly lower in the lung tissue than in control mice and the number of neutrophils in the airways of depleted mice was negligible (S6 Fig). A more likely explanation is that CD8^+^ T cells adapt to utilize a CXCL-12 independent mode of migration through the tissue or that a secondary wave of CXCL-12 independent CD8^+^ T cells infiltrates the lungs, possibly in response to the increased viral load. It may reflect the fact that the clonal burst of T cells in the draining lymph nodes is not directly affected by the elimination of neutrophils and given more time, the T cells accumulate to similar numbers by a later time point. Supporting this interpretation, mice that lack neutrophil derived CXCL-12 display early defects in CD8^+^ T cell responses that recover by day 9 post-infection[35]. This recovery phenomenon was also seen in the context of adoptive transfer of very few or large numbers of T cells[46]. Although changes are observed early after transfer, these differences are abated by day 10 post-infection.

With a concordant delay in viral clearance and the appearance of a robust effector T cell response in the absence of neutrophils, we predicted that T cell migration kinetics and phenotypes would reflect these changes. Our lab has established a tracheitis model for examining virus-specific CD8^+^ T cells during influenza virus infection using multiphoton microscopy[37]. Using this model, we identified increases in velocity and displacement rates of the CD8^+^ T cells in mice deficient in neutrophils at day 9 post-infection, a time at which virus has been cleared in control mice. Cells at this time are also phenotypically distinct, and have a larger, less round appearance. Both the measured parameters and phenotype are indicative of cells that are actively migrating and surveying surrounding tissue, compared with control cells, which are more limited in the distance traveled. Interestingly, by day 10 post-infection, the parameters measured in depleted mice have similar features to those from day 9 of control mice, suggesting that the prolonged virus replication and/or antigen availability alters CD8^+^ T cell behavior. These observations further support the notion that the CD8^+^ T cell response is not fully impaired, but rather delayed.

Given that the CD8^+^ T cell response recovered to, at minimum, normal levels, it was not surprising that we were able to detect comparable numbers of phenotypic T_RM_ cells in the airways of both groups of mice. However, this was inconsistent with our previous report showing that NP tetramer^+^ T cells were present in a reduced capacity in tracheal and lung tissue[35]. There are a number of differences in how this question was approached, which could lead to the observed changes. At the day 50 time point previously evaluated, there could still be the presence of effector cells in the neutrophil competent mice which are creating the discrepancy. Furthermore, neither of our studies sought to evaluate the populations of memory T cells that are known to be located in vascular walls[47,48], which could contribute to differences observed. It is unclear whether these cells depend on neutrophil signals and the overall relative contributions of BAL versus vascular memory have not been distinguished. However, our group and others have shown that the cells localized to the airways are required for protection from heterosubtypic challenge, despite expressing lower levels of LFA-1[4,5,41]. Our current study also performed a more extensive analysis of the cells within the CD8^+^ T cell compartment, focusing not only on tetramer positive cells, but also evaluating the expression of prototypical T_RM_ markers including integrins α1 and αE, and S1P1 inhibitor, CD69. Our data suggest that the T_RM_ precursor cells, which have yet to be concretely identified, may exhibit different signals and chemokine requirements than their early effector counterparts, which are affected by the absence of neutrophils.

Importantly, we not only demonstrated the presence of cells that phenotypically comprise a T_RM_ population, but we showed that the cells elicit similar effector features when stimulated *in vitro*. In both groups of mice, cells can produce IFNγ and TNFα in response to stimulus. This is important given that IFNγ has been specifically labeled as essential for a functional resident memory response, and cells deficient in the cytokine are not protective[41]. Additionally, CD8^+^ T_RM_ cells degranulated upon restimulation, however, granzyme B was detected at very low levels. The cells may have rapidly degranulated cellular stores too early for our detection, they may be releasing other granule associated proteins such as granzyme A, which we observed *in vitro*, or they may degranulate defective non-cytolytic or empty granules. Central memory cells display similar features upon reactivation, staining for Lamp-1 in the absence of granzyme B, and were shown to rapidly deplete stores[49]. The result is cells that appear negative for granzyme B for a period during which the cell replenishes the granzyme stores. It is also possible that T_RM_ cells are lacking in cytolytic mediators as other studies have shown them to be less effective in killing target cells *in vitro*[41].

Lastly, and critically, our data reveal that mice infected with influenza virus in the absence of neutrophils are protected from secondary challenge with a lethal dose of a heterosubtypic virus. All mice with previous virus exposure survived this insult and displayed similar weight loss and viral loads, whereas antigen inexperienced mice rapidly succumbed to infection. This further indicates that T_RM_ cells generated in the absence of neutrophils are similarly equipped to fight infection, but that in the absence of the T_RM_ population, mice become vulnerable to future insults.

## Supporting Information

S1 Fig Treatment with anti-Ly6G antibody (clone 1A8) reduces neutrophil population in the lung by greater than 90%. Neutrophil depletion regimen (A). Neutrophil populations in the lungs were examined at day 6 post-infection by flow cytometry (B) and quantified (C). Cytospins were used to further verify depletion at the same time point (D). Data is representative of 3 separate experiments. * p-value <0.05 by Student’s T test.
(TIF)

S2 Fig Neutrophil depletion does not alter the development of a phenotypic T_RM_ population in the lung tissue. Cells from digested lung tissue after lavage from influenza naïve (uninfected = UI), IgG Control during primary X31 infection (Control = IgG), and Neutrophil Depleted during primary X31 infection (Neutrophil Depleted = ND) were analyzed by flow cytometry for the whole CD8^+^ T cell population and the following CD8^+^ T cell subsets: NP tetramer^+^, CD49a/CD103, and CD103/CD69. Data is a compilation of 3 separate experiments and represented as mean ± SEM.
(TIF)

S3 Fig At 3 months post-infection, the lung CD8^+^ T cell population is more diverse. CD8^+^ T cells from lung tissue and BAL were stained with CD62L and CD44 to define different subsets of T cells that remain in their respective compartment after infection. Data shown is representative of 3 separate experiments.
(TIF)

S4 Fig CD8^+^ T cells in the lung parenchyma display similar functions in vitro regardless of prior neutrophil status. Lung cells IgG Control and Neutrophil Depleted mice at 3 months post-infection were stimulated with NP peptide in vitro for 6 hours with BFA for the last 4 hours. Cells were analyzed for production of IFNγ, TNFα, Lamp1, Granzyme B, and Granzyme A. Based off of cell counts prior to culturing, total positive cells were quantified.
(TIF)

S5 Fig CD8^+^ T cell populations in the lung tissue at days 2 and 6 post-rechallenge. Representative flow plots of CD8^+^ T cells derived from the BAL to evaluate NP-specificity and expression of CD49a/CD103 or CD103/CD69 at days 2 and 6 post-infection. Mice with no history of influenza virus (No prime), primary X31 with IgG control antibody (IgG Control X31 Prime) and primary X31 with Neutrophil Depletion (Neut. Depletion X31 Prime) were the 3 groups evaluated at day 2. Only mice with a history of influenza virus infection (IgG Control X31 Prime and Neut. Depletion X31 Prime) were examined at day 6, due to the susceptibility and mortality of naive mice. Data shown are a concatenation of 3 mice.
(TIF)

S6 Fig Mice depleted of neutrophils during primary influenza virus infection maintain significantly lower levels of neutrophils in the lung and BAL through day 14. Mice infected with HK-X31 influenza virus with and without neutrophil depletion were examined for neutrophils at day 14 post-infection in the BAL and lung tissue. Neutrophils were identified as cells expressing high levels of both Gr-1 and CD11b. Data are representative of 3 separate experiments. *p<0.05 by Student’s T test.
(TIF)

S1 Video GFP^+^OT-1 CD8^+^ T cells shown in green in the trachea of a control mouse at day 9 post-infection with HK-X31 OVA virus. Video is displayed in extended focus at 256 pixel resolution at 25X magnification.
(AVI)

S2 Video GFP^+^OT-1 CD8^+^ T cells in green in the trachea of a neutrophil depleted mouse at day 9 post-infection with HK-X31 OVA virus. Video is shown in extended focus at 256 pixel resolution at 25X magnification.
(AVI)

## References

[1] Bevan MJ. Memory T cells as an occupying force. Eur J Immunol. 2011;41: 1192–1195. 10.1002/eji.201041377 21469134 PMC3304501

[2] Gebhardt T, Whitney PG, Zaid A, Mackay LK, Brooks AG, Heath WR, et al Different patterns of peripheral migration by memory CD4+ and CD8+ T cells. Nature. 2011;477: 216–219. 10.1038/nature10339 21841802

[3] Schenkel JM, Fraser KA, Beura LK, Pauken KE, Vezys V, Masopust D. T cell memory. Resident memory CD8 T cells trigger protective innate and adaptive immune responses. Science. 2014;346: 98–101. 10.1126/science.1254536 25170049 PMC4449618

[4] Wu T, Hu Y, Lee Y-T, Bouchard KR, Benechet A, Khanna K, et al Lung-resident memory CD8 T cells (TRM) are indispensable for optimal cross-protection against pulmonary virus infection. J Leukoc Biol. 2014;95: 215–224. 10.1189/jlb.0313180 24006506 PMC3896663

[5] Ray SJ, Franki SN, Pierce RH, Dimitrova S, Koteliansky V, Sprague AG, et al The collagen binding alpha1beta1 integrin VLA-1 regulates CD8 T cell-mediated immune protection against heterologous influenza infection. Immunity. 2004;20: 167–179. 14975239 10.1016/s1074-7613(04)00021-4

[6] Gebhardt T, Wakim LM, Eidsmo L, Reading PC, Heath WR, Carbone FR. Memory T cells in nonlymphoid tissue that provide enhanced local immunity during infection with herpes simplex virus. Nat Immunol. 2009;10: 524–530. 10.1038/ni.1718 19305395

[7] Masopust D, Choo D, Vezys V, Wherry EJ, Duraiswamy J, Akondy R, et al Dynamic T cell migration program provides resident memory within intestinal epithelium. J Exp Med. 2010;207: 553–564. 10.1084/jem.20090858 20156972 PMC2839151

[8] Wakim LM, Woodward-Davis A, Bevan MJ. Memory T cells persisting within the brain after local infection show functional adaptations to their tissue of residence. Proc Natl Acad Sci USA. 2010;107: 17872–17879. 10.1073/pnas.1010201107 20923878 PMC2964240

[9] Hofmann M, Pircher H. E-cadherin promotes accumulation of a unique memory CD8 T- cell population in murine salivary glands. Proc Natl Acad Sci USA. 2011;108: 16741–16746. 10.1073/pnas.1107200108 21930933 PMC3189029

[10] Schenkel JM, Fraser KA, Vezys V, Masopust D. Sensing and alarm function of resident memory CD8^+^ T cells. Nat Immunol. 2013;14: 509–513. 10.1038/ni.2568 23542740 PMC3631432

[11] Klonowski KD, Williams KJ, Marzo AL, Blair DA, Lingenheld EG, Lefrançois L. Dynamics of blood-borne CD8 memory T cell migration in vivo. Immunity. 2004;20: 551–562. 15142524 10.1016/s1074-7613(04)00103-7

[12] Wiley JA, Hogan RJ, Woodland DL, Harmsen AG. Antigen-specific CD8(+) T cells persist in the upper respiratory tract following influenza virus infection. J Immunol. 2001;167: 3293–3299. 11544317 10.4049/jimmunol.167.6.3293

[13] Berlin C, Berg EL, Briskin MJ, Andrew DP, Kilshaw PJ, Holzmann B, et al Alpha 4 beta 7 integrin mediates lymphocyte binding to the mucosal vascular addressin MAdCAM-1. Cell. 1993;74: 185–195. 7687523 10.1016/0092-8674(93)90305-a

[14] Zabel BA, Agace WW, Campbell JJ, Heath HM, Parent D, Roberts AI, et al Human G protein-coupled receptor GPR-9-6/CC chemokine receptor 9 is selectively expressed on intestinal homing T lymphocytes, mucosal lymphocytes, and thymocytes and is required for thymus-expressed chemokine-mediated chemotaxis. J Exp Med. 1999;190: 1241–1256. 10544196 10.1084/jem.190.9.1241 PMC2195678

[15] Kunkel EJ, Campbell JJ, Haraldsen G, Pan J, Boisvert J, Roberts AI, et al Lymphocyte CC chemokine receptor 9 and epithelial thymus-expressed chemokine (TECK) expression distinguish the small intestinal immune compartment: Epithelial expression of tissue- specific chemokines as an organizing principle in regional immunity. J Exp Med. 2000;192: 761–768. 10974041 10.1084/jem.192.5.761 PMC2193265

[16] Campbell JJ, Haraldsen G, Pan J, Rottman J, Qin S, Ponath P, et al The chemokine receptor CCR4 in vascular recognition by cutaneous but not intestinal memory T cells. Nature. 1999;400: 776–780. 10.1038/23495 10466728

[17] Fuhlbrigge RC, Kieffer JD, Armerding D, Kupper TS. Cutaneous lymphocyte antigen is a specialized form of PSGL-1 expressed on skin-homing T cells. Nature. 1997;389: 978–981. 10.1038/40166 9353122

[18] Clark RA, Chong B, Mirchandani N, Brinster NK, Yamanaka K-I, Dowgiert RK, et al The vast majority of CLA+ T cells are resident in normal skin. J Immunol. 2006;176: 4431–4439. 16547281 10.4049/jimmunol.176.7.4431

[19] Adachi T, Kobayashi T, Sugihara E, Yamada T, Ikuta K, Pittaluga S, et al Hair follicle- derived IL-7 and IL-15 mediate skin-resident memory T cell homeostasis and lymphoma. Nat Med. 2015;21: 1272–1279. 10.1038/nm.3962 26479922 PMC4636445

[20] Slütter B, Pewe LL, Kaech SM, Harty JT. Lung airway-surveilling CXCR3(hi) memory CD8(+) T cells are critical for protection against influenza A virus. Immunity. 2013;39: 939–948. 10.1016/j.immuni.2013.09.013 24238342 PMC3872058

[21] Lu B, Humbles A, Bota D, Gerard C, Moser B, Soler D, et al Structure and function of the murine chemokine receptor CXCR3. Eur J Immunol. 1999;29: 3804–3812. doi: 10.1002/(SICI)1521-4141(199911)29:11<3804::AID- IMMU3804>3.0.CO;2–9 10556837 10.1002/(SICI)1521-4141(199911)29:11<3804::AID-IMMU3804>3.0.CO;2-9

[22] Kohlmeier JE, Reiley WW, Perona-Wright G, Freeman ML, Yager EJ, Connor LM, et al Inflammatory chemokine receptors regulate CD8(+) T cell contraction and memory generation following infection. J Exp Med. 2011;208: 1621–1634. 10.1084/jem.20102110 21788409 PMC3149221

[23] Kurachi M, Kurachi J, Suenaga F, Tsukui T, Abe J, Ueha S, et al Chemokine receptor CXCR3 facilitates CD8(+) T cell differentiation into short-lived effector cells leading to memory degeneration. J Exp Med. 2011;208: 1605–1620. 10.1084/jem.20102101 21788406 PMC3149224

[24] Mackay LK, Rahimpour A, Ma JZ, Collins N, Stock AT, Hafon M-L, et al The developmental pathway for CD103(+)CD8+ tissue-resident memory T cells of skin. Nat Immunol. 2013;14: 1294–1301. 10.1038/ni.2744 24162776

[25] Lee Y-T, Suarez-Ramirez JE, Wu T, Redman JM, Bouchard K, Hadley GA, et al Environmental and antigen receptor-derived signals support sustained surveillance of the lungs by pathogen-specific cytotoxic T lymphocytes. J Virol. 2011;85: 4085–4094. 10.1128/JVI.02493-10 21345961 PMC3126261

[26] Cepek KL, Shaw SK, Parker CM, Russell GJ, Morrow JS, Rimm DL, et al Adhesion between epithelial cells and T lymphocytes mediated by E-cadherin and the alpha E beta 7 integrin. Nature. 1994;372: 190–193. 10.1038/372190a0 7969453

[27] Matloubian M, Lo CG, Cinamon G, Lesneski MJ, Xu Y, Brinkmann V, et al Lymphocyte egress from thymus and peripheral lymphoid organs is dependent on S1P receptor 1. Nature. 2004;427: 355–360. 10.1038/nature02284 14737169

[28] Shiow LR, Rosen DB, Brdicková N, Xu Y, An J, Lanier LL, et al CD69 acts downstream of interferon-alpha/beta to inhibit S1P1 and lymphocyte egress from lymphoid organs. Nature. 2006;440: 540–544. 10.1038/nature04606 16525420

[29] Skon CN, Lee J-Y, Anderson KG, Masopust D, Hogquist KA, Jameson SC. Transcriptional downregulation of S1pr1 is required for the establishment of resident memory CD8+ T cells. Nat Immunol. 2013;14: 1285–1293. 10.1038/ni.2745 24162775 PMC3844557

[30] Richter M, Ray SJ, Chapman TJ, Austin SJ, Rebhahn J, Mosmann TR, et al Collagen distribution and expression of collagen-binding alpha1beta1 (VLA-1) and alpha2beta1 (VLA-2) integrins on CD4 and CD8 T cells during influenza infection. J Immunol. 2007;178: 4506–4516. 17372009 10.4049/jimmunol.178.7.4506

[31] Miner JH, Sanes JR. Collagen IV alpha 3, alpha 4, and alpha 5 chains in rodent basal laminae: sequence, distribution, association with laminins, and developmental switches. J Cell Biol. 1994;127: 879–891. 7962065 10.1083/jcb.127.3.879 PMC2120241

[32] Naik S, Bouladoux N, Linehan JL, Han S-J, Harrison OJ, Wilhelm C, et al Commensal- dendritic-cell interaction specifies a unique protective skin immune signature. Nature. 2015;520: 104–108. 10.1038/nature14052 25539086 PMC4667810

[33] Zhang N, Bevan MJ. Transforming growth factor-β signaling controls the formation and maintenance of gut-resident memory T cells by regulating migration and retention. Immunity. 2013;39: 687–696. 10.1016/j.immuni.2013.08.019 24076049 PMC3805703

[34] Liang S, Mozdzanowska K, Palladino G, Gerhard W. Heterosubtypic immunity to influenza type A virus in mice. Effector mechanisms and their longevity. J Immunol. 1994;152: 1653–1661. 8120375

[35] Lim K, Hyun Y-M, Lambert-Emo K, Capece T, Bae S, Miller R, et al Neutrophil trails guide influenza-specific CD8^+^ T cells in the airways. Science. 2015;349: aaa4352. 10.1126/science.aaa4352 26339033 PMC4809646

[36] Tate MD, Brooks AG, Reading PC, Mintern JD. Neutrophils sustain effective CD8(+) T- cell responses in the respiratory tract following influenza infection. Immunol Cell Biol. 2012;90: 197–205. 10.1038/icb.2011.26 21483446

[37] Lambert-Emo K, Hyun Y-M, Reilly E, Barilla C, Gerber S, Fowell D, et al Live Imaging of Influenza Infection of the Trachea Reveals Dynamic Regulation of CD8+ T Cell Motility by Antigen. PLoS Pathog. 2016. 10.1371/journal.ppat.1005881 PMC5028057 27644089

[38] Belz GT, Xie W, Doherty PC. Diversity of epitope and cytokine profiles for primary and secondary influenza a virus-specific CD8+ T cell responses. J Immunol. 2001;166: 4627–4633. 11254721 10.4049/jimmunol.166.7.4627

[39] Tate MD, Deng Y-M, Jones JE, Anderson GP, Brooks AG, Reading PC. Neutrophils ameliorate lung injury and the development of severe disease during influenza infection. J Immunol. 2009;183: 7441–7450. 10.4049/jimmunol.0902497 19917678

[40] Mrass P, Kinjyo I, Ng LG, Reiner SL, Puré E, Weninger W. CD44 mediates successful interstitial navigation by killer T cells and enables efficient antitumor immunity. Immunity. 2008;29: 971–985. 10.1016/j.immuni.2008.10.015 19100702 PMC2757129

[41] McMaster SR, Wilson JJ, Wang H, Kohlmeier JE. Airway-Resident Memory CD8 T Cells Provide Antigen-Specific Protection against Respiratory Virus Challenge through Rapid IFN-γ Production. J Immunol. 2015;195: 203–209. 10.4049/jimmunol.1402975 26026054 PMC4475417

[42] Topham DJ, Tripp RA, Doherty PC. CD8+ T cells clear influenza virus by perforin or Fas-dependent processes. J Immunol. 1997;159: 5197–5200. 9548456

[43] Webby RJ, Andreansky S, Stambas J, Rehg JE, Webster RG, Doherty PC, et al Protection and compensation in the influenza virus-specific CD8+ T cell response. Proc Natl Acad Sci USA. 2003;100: 7235–7240. 10.1073/pnas.1232449100 12775762 PMC165859

[44] Furze RC, Rankin SM. Neutrophil mobilization and clearance in the bone marrow. Immunology. 2008;125: 281–288. 10.1111/j.1365-2567.2008.02950.x 19128361 PMC2669132

[45] Kreisel D, Nava RG, Li W, Zinselmeyer BH, Wang B, Lai J, et al In vivo two-photon imaging reveals monocyte-dependent neutrophil extravasation during pulmonary inflammation. Proc Natl Acad Sci USA. 2010;107: 18073–18078. 10.1073/pnas.1008737107 20923880 PMC2964224

[46] Powell TJ, Brown DM, Hollenbaugh JA, Charbonneau T, Kemp RA, Swain SL, et al CD8+ T cells responding to influenza infection reach and persist at higher numbers than CD4+ T cells independently of precursor frequency. Clin Immunol. 2004;113: 89–100. 10.1016/j.clim.2004.05.006 15380534

[47] Anderson KG, Sung H, Skon CN, Lefrançois L, Deisinger A, Vezys V, et al Cutting edge: intravascular staining redefines lung CD8 T cell responses. J Immunol. 2012;189: 2702–2706. 10.4049/jimmunol.1201682 22896631 PMC3436991

[48] Wakim LM, Gupta N, Mintern JD, Villadangos JA. Enhanced survival of lung tissue- resident memory CD8^+^ T cells during infection with influenza virus due to selective expression of IFITM3. Nat Immunol. 2013;14: 238–245. 10.1038/ni.2525 23354485

[49] Wolint P, Betts MR, Koup RA, Oxenius A. Immediate cytotoxicity but not degranulation distinguishes effector and memory subsets of CD8+ T cells. J Exp Med. 2004;199: 925–936. doi: 10.1084/jem.20031799 667 15051762 10.1084/jem.20031799 PMC2211884

