# Supplementary figures and images for: The Effects of Acute Neutrophil Depletion on Resolution of Acute Influenza Infection, Establishment of Tissue Resident Memory (TRM), and Heterosubtypic Immunity
Source: PLoS One. 2016 Oct 14;11(10):e0164247. doi: 10.1371/journal.pone.0164247 (PMC5065200; doi:10.1371/journal.pone.0164247)

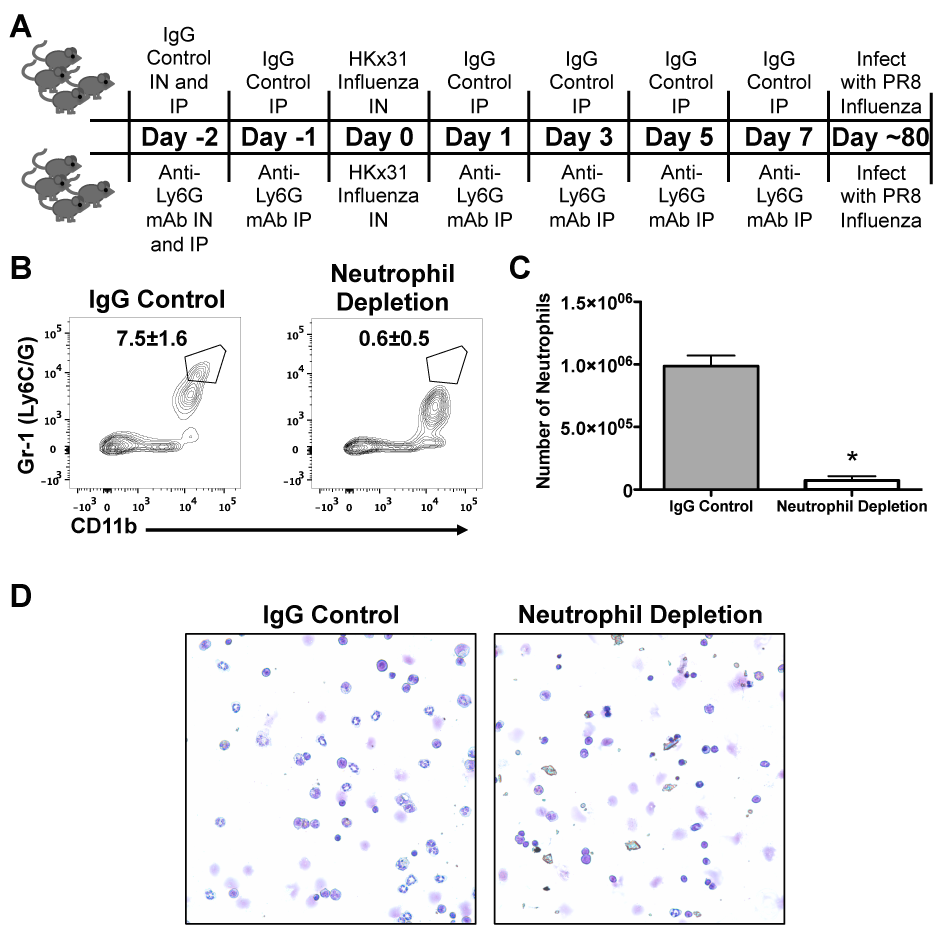

Supplement: S1 Fig — Neutrophil depletion regimen (A). Neutrophil populations in the lungs were examined at day 6 post-infection by flow cytometry (B) and quantified (C). Cytospins were used to further verify depletion at the same time point (D). Data is representative of 3 separate experiments. * p-value <0.05 by Student’s T test. (TIF) [file pone.0164247.s001.tif]

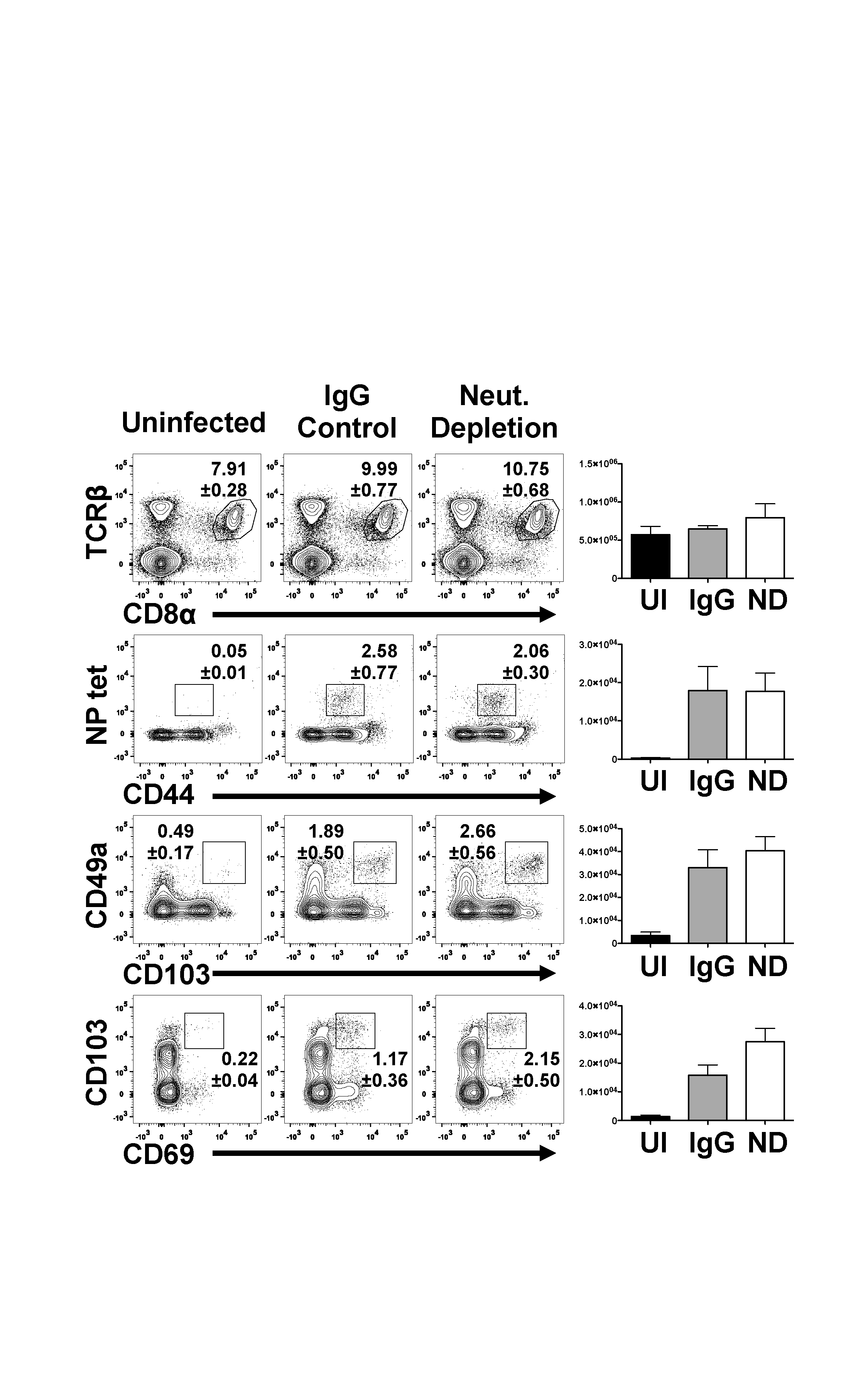

Supplement: S2 Fig — Cells from digested lung tissue after lavage from influenza naïve (uninfected = UI), IgG Control during primary X31 infection (Control = IgG), and Neutrophil Depleted during primary X31 infection (Neutrophil Depleted = ND) were analyzed by flow cytometry for the whole CD8+ T cell population and the following CD8+ T cell subsets: NP tetramer+, CD49a/CD103, and CD103/CD69. Data is a compilation of 3 separate experiments and represented as mean ± SEM. (TIF) [file pone.0164247.s002.tif]

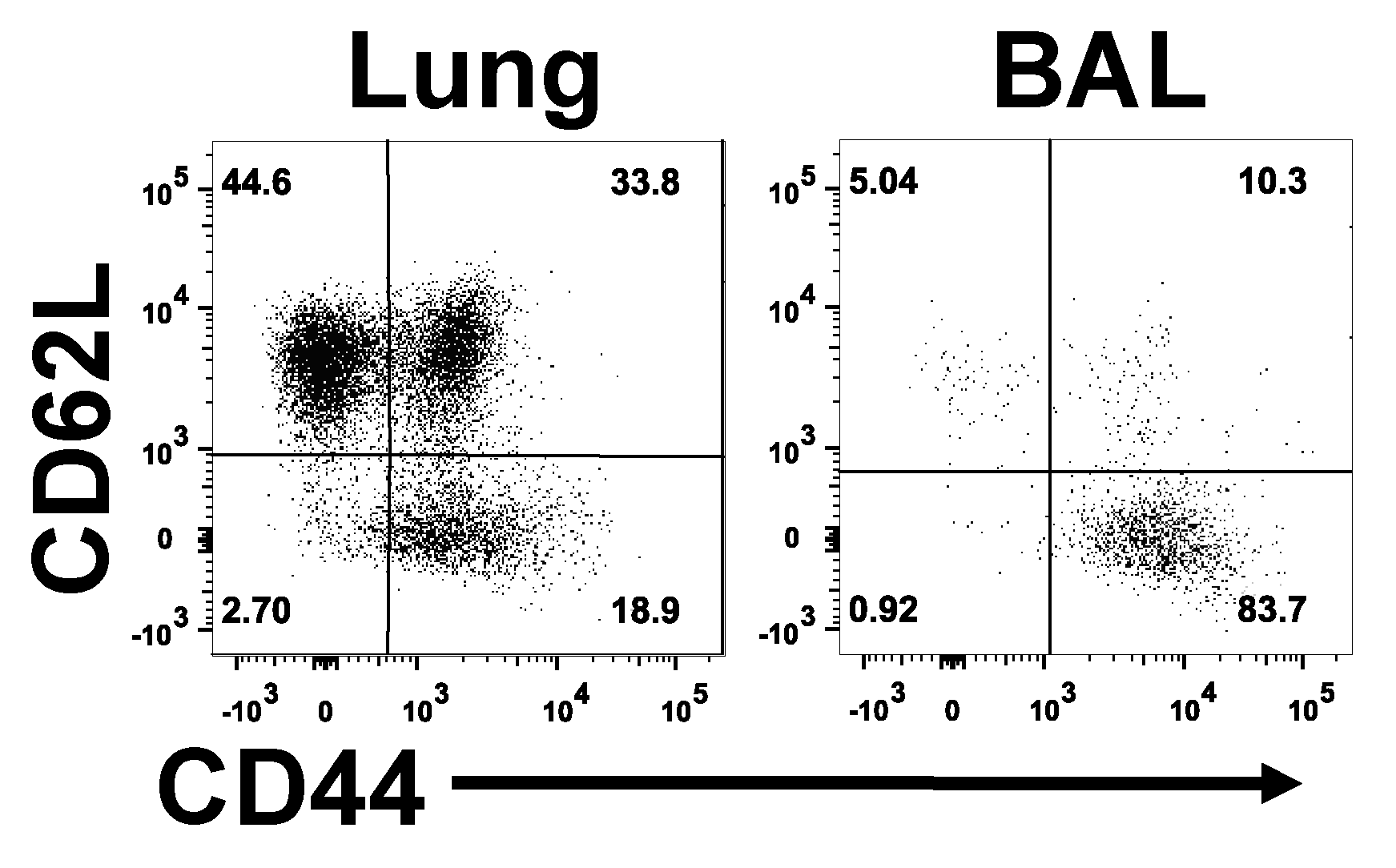

Supplement: S3 Fig — CD8+ T cells from lung tissue and BAL were stained with CD62L and CD44 to define different subsets of T cells that remain in their respective compartment after infection. Data shown is representative of 3 separate experiments. (TIF) [file pone.0164247.s003.tif]

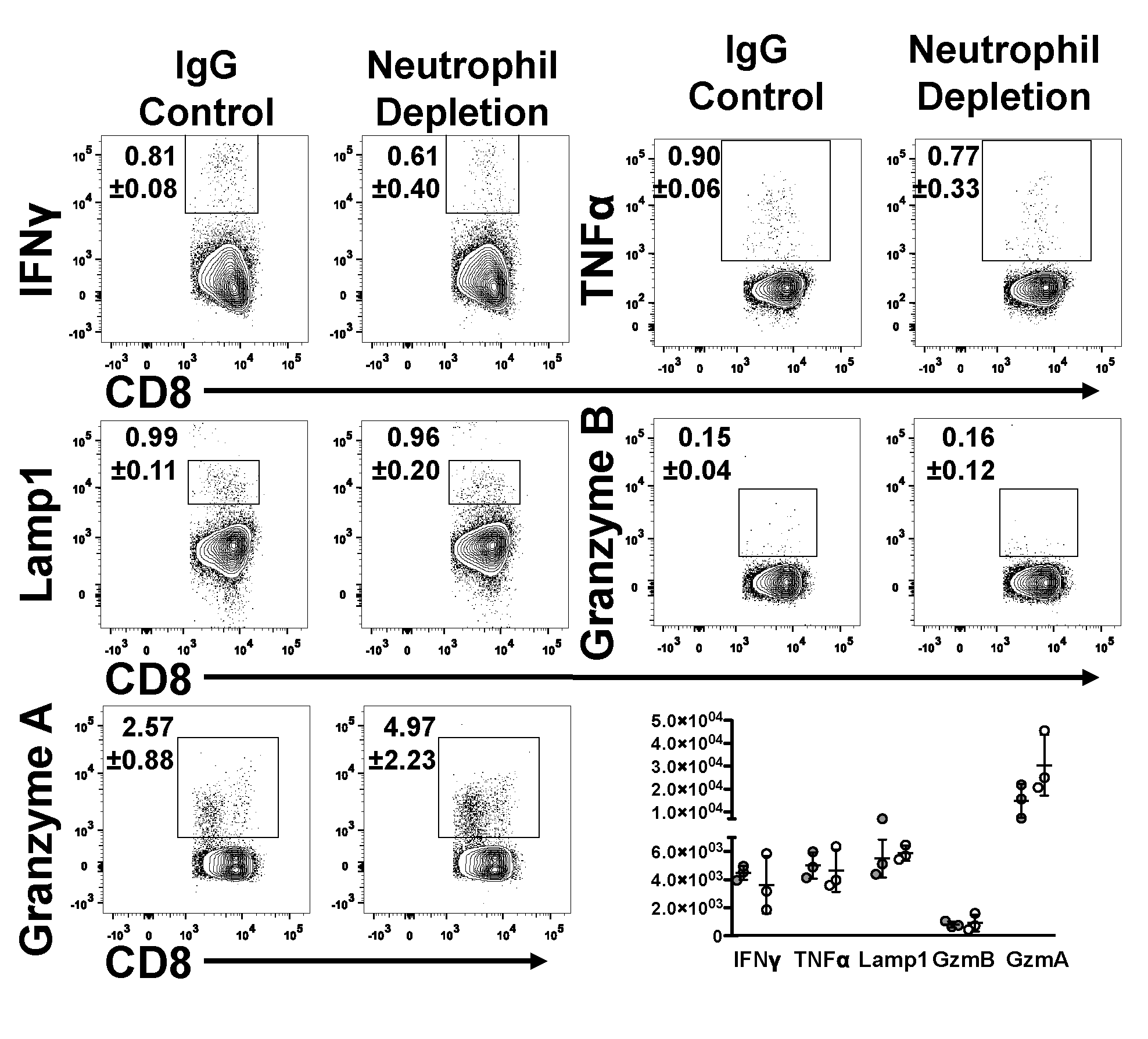

Supplement: S4 Fig — Lung cells IgG Control and Neutrophil Depleted mice at 3 months post-infection were stimulated with NP peptide in vitro for 6 hours with BFA for the last 4 hours. Cells were analyzed for production of IFNγ, TNFα, Lamp1, Granzyme B, and Granzyme A. Based off of cell counts prior to culturing, total positive cells were quantified. (TIF) [file pone.0164247.s004.tif]

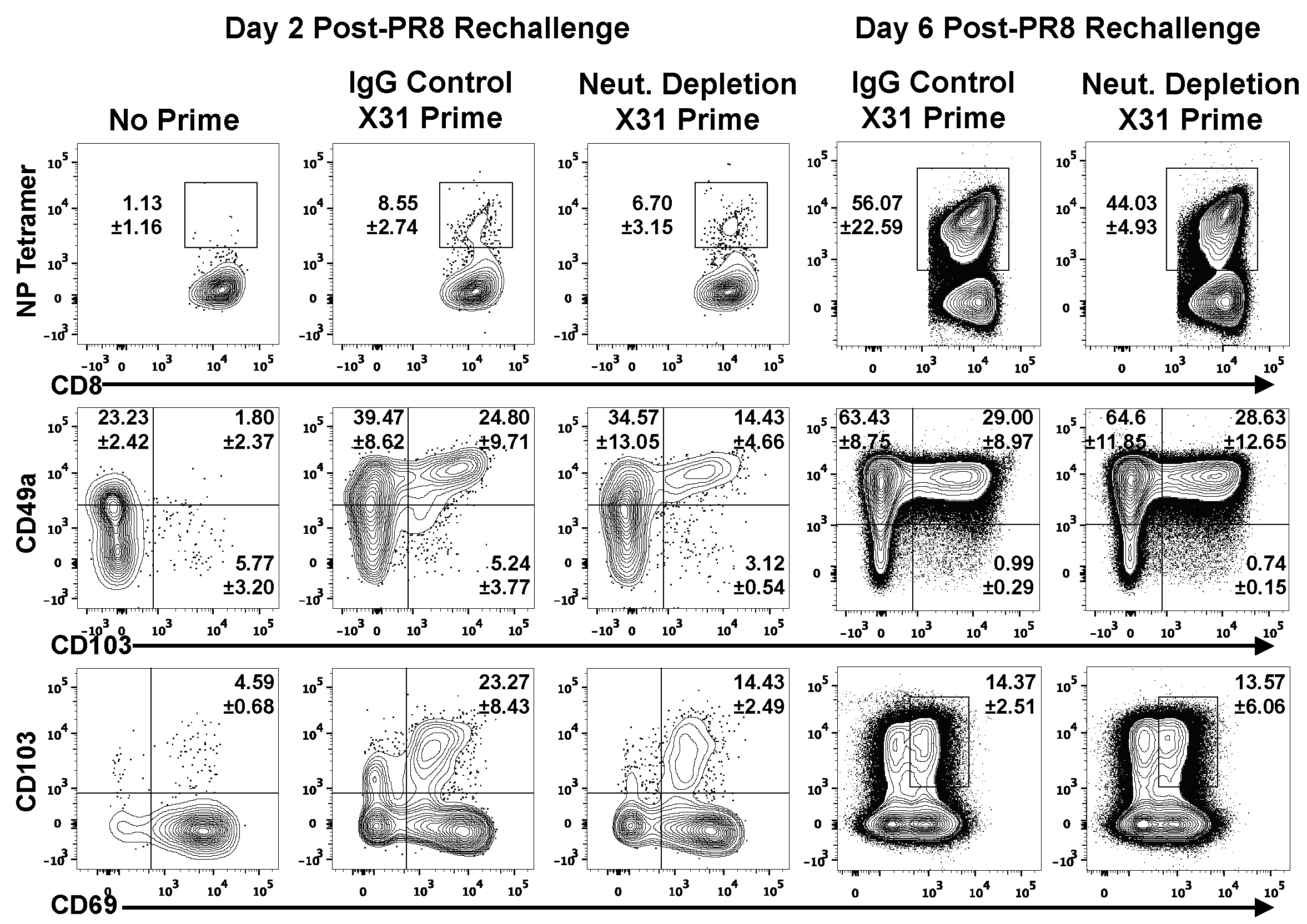

Supplement: S5 Fig — Representative flow plots of CD8+ T cells derived from the BAL to evaluate NP-specificity and expression of CD49a/CD103 or CD103/CD69 at days 2 and 6 post-infection. Mice with no history of influenza virus (No prime), primary X31 with IgG control antibody (IgG Control X31 Prime) and primary X31 with Neutrophil Depletion (Neut. Depletion X31 Prime) were the 3 groups evaluated at day 2. Only mice with a history of influenza virus infection (IgG Control X31 Prime and Neut. Depletion X31 Prime) were examined at day 6, due to the susceptibility and mortality of naive mice. Data shown are a concatenation of 3 mice. (TIF) [file pone.0164247.s005.tif]

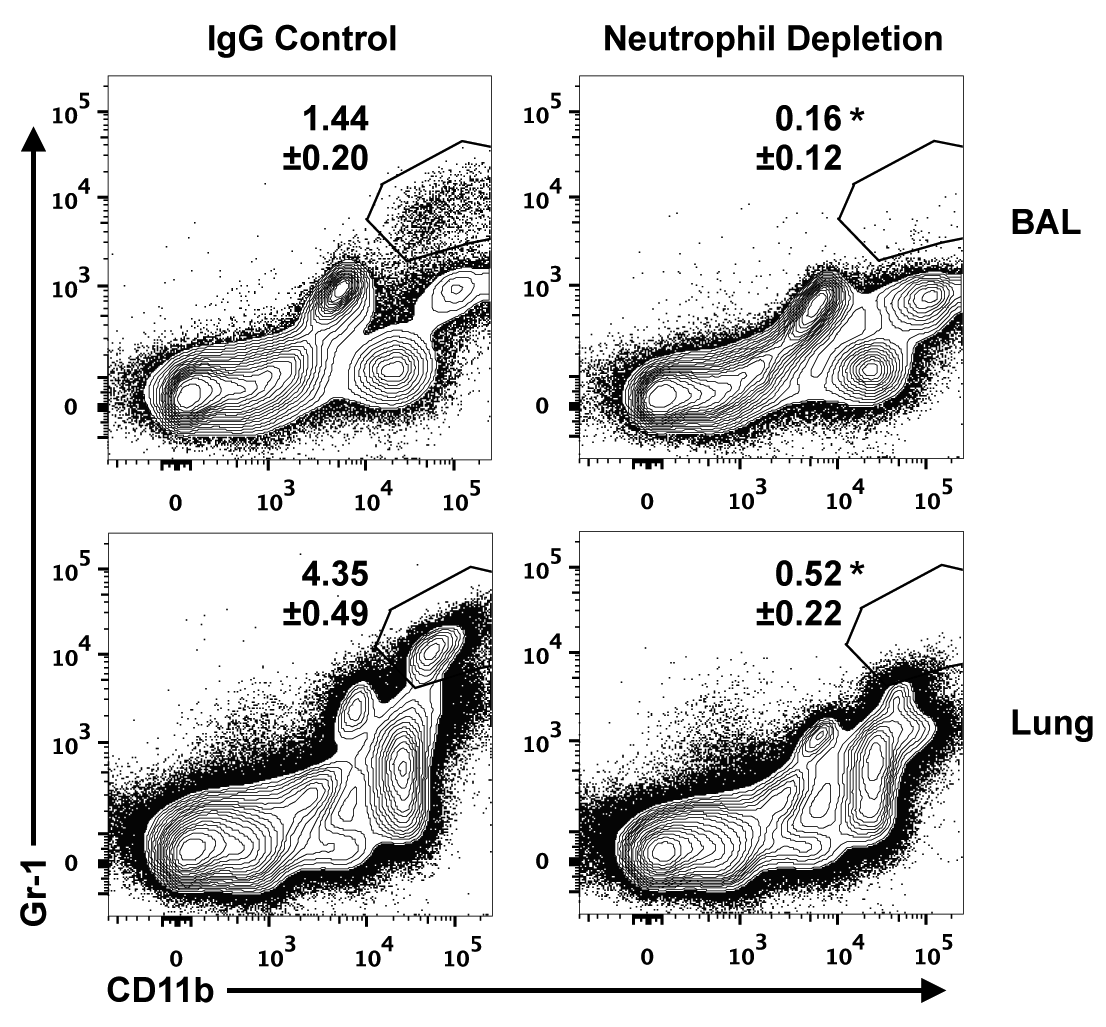

Supplement: S6 Fig — Mice infected with HK-X31 influenza virus with and without neutrophil depletion were examined for neutrophils at day 14 post-infection in the BAL and lung tissue. Neutrophils were identified as cells expressing high levels of both Gr-1 and CD11b. Data are representative of 3 separate experiments. *p<0.05 by Student’s T test. (TIF) [file pone.0164247.s006.tif]
